# Body Weight Support Combined With Treadmill in the Rehabilitation of Parkinsonian Gait: A Review of Literature and New Data From a Controlled Study

**DOI:** 10.3389/fneur.2018.01066

**Published:** 2019-02-08

**Authors:** Eliana Berra, Roberto De Icco, Micol Avenali, Carlotta Dagna, Silvano Cristina, Claudio Pacchetti, Mauro Fresia, Giorgio Sandrini, Cristina Tassorelli

**Affiliations:** ^1^Neurorehabilitation Unit and Parkinson Unit, IRCCS Mondino Foundation, Pavia, Italy; ^2^Department of Brain and Behavioral Sciences, University of Pavia, Pavia, Italy

**Keywords:** body weight support treadmill training, gait rehabilitation, computerized gait analysis, Parkinson's disease, neurorehabilitation

## Abstract

**Background:** Gait disorders represent disabling symptoms in Parkinson's Disease (PD). The effectiveness of rehabilitation treatment with Body Weight Support Treadmill Training (BWSTT) has been demonstrated in patients with stroke and spinal cord injuries, but limited data is available in PD.

**Aims:** The aim of the study is to investigate the efficacy of BWSTT in the rehabilitation of gait in PD patients.

**Methods:** Thirty-six PD inpatients were enrolled and performed rehabilitation treatment for 4-weeks, with daily sessions. Subjects were randomly divided into two groups: both groups underwent daily 40-min sessions of traditional physiokinesitherapy followed by 20-min sessions of overground gait training (Control group) or BWSTT (BWSTT group). The efficacy of BWSTT was evaluated with clinical scales and Computerized Gait Analysis (CGA). Patients were tested at baseline (T0) and at the end of the 4-weeks rehabilitation period (T1).

**Results:** Both BWSTT and Control groups experienced a significant improvement in clinical scales as FIM and UPDRS and in gait parameters for both interventions. Even if we failed to detect any statistically significant differences between groups in the different clinical and gait parameters, the intragroup analysis captured a specific pattern of qualitative improvement associated to cadence and stride duration for the BWSTT group and to the swing/stance ratio for the Control group. Four patients with chronic pain or anxious symptoms did not tolerate BWSTT.

**Conclusions:** BWSTT and traditional rehabilitation treatment are both effective in improving clinical motor functions and kinematic gait parameters. BWSTT may represent an option in PD patients with specific symptoms that limit traditional overground gait training, e.g., severe postural instability, balance disorder, orthostatic hypotension. BWSTT is generally well-tolerated, though caution is needed in subjects with chronic pain or with anxious symptoms.

**Clinical Trial Registration:** www.ClinicalTrials.gov, identifier: NCT03815409

## Introduction

Gait disorders in Parkinson's Disease (PD) are due to dopaminergic nigrostriatal pathways degeneration and represent important components of the disability (1).

In PD, gait is characterized by a significant reduction of stride length (2). Inadequate flexion at the ankle and knee, reduction of heel strike, forward-flexed trunk, reduced arm swing with asymmetric stride times for lower limbs and significant stride-to-stride variability are frequently associated (3–8).

The efficacy of pharmacological treatment with Levodopa is frequently uncomplete (9) and adjuvant rehabilitation treatment is recommended. Body weight supported treadmill training (BWSTT) represents a promising rehabilitative approach for gait impairment in PD (10, 11). Effectiveness of BWSTT on gait, balance and motor function has been demonstrated in different neurological diseases, especially in stroke (12) and spinal cord injury (13). In stroke, authors reported that BWSTT appears as a safe method of training, providing a major sense of security regarding falls and facilitating free leg movements, compared with treadmill alone (12). In addition, stroke patients treated with BWSTT were able to walk for a longer duration and with a minimal increase in heart rates (14). In PD patients, BWSTT has been tested in small controlled studies that have suggested a clinically detectable beneficial effect (10, 11). BWSTT seems also effective in improving balance in PD (15–17), evaluated both with clinical scales (as Berg Balance Scale, BBS) and dynamic posturography (18). In PD, many data in literature show how treadmill training, acting as a sensory cue, improves kinetic and kinematic parameters, studied with computerized gait analysis (CGA), more than physiotherapy alone. Toole and Ganesan highlighted a positive impact of BWSTT on postural instability using instrumental investigations (15, 18). Regarding gait rehabilitation, most of the data recorded with computerized movement analysis derived from BWSTT delivered with robotic devices (19–22). Only a limited number of studies has instead investigated the effect of non-robotic BWSTT on gait kinetic and kinematic data. Ganesan et al. used an instrumental evaluation, but it must be noted that they recorded kinematic gait parameters during a treadmill-assisted walk, which prevents generalization to overground gait (23). Another study (24) showed an improvement in gait speed and cadence after a single session of BWSTT both in PD patients and healthy controls, but did not provide any data on the effect of multiple sessions or on any possible retention of the effect.

In this manuscript we present a revision of the main literature on gait rehabilitation in PD using BWSTT with and without robotic devices, and the findings of a randomized, controlled, parallel-group study conducted on a representative sample of PD subjects to investigate the efficacy of BWSTT using the computerized gait analysis for the precise definition of qualitative and quantitative effects.

### Related Works

#### BWS Delivered Without Robotic Devices

The first report of BWSTT efficacy in gait rehabilitation of PD belongs to Miyai et al. Ten patients with PD were enrolled in a cross-over study and treated for 4 consecutive weeks with BWSTT (20% of unweighting for 12 min followed by another 12-min period of 10% of unweighting) or conventional physical therapy (CPT). The Authors showed that BWSTT was superior to CPT in improving gait disturbances and disability at the end of the rehabilitative period. More specifically BWSTT proved superior to CPT in improving UPDRS scores, gait speed and stride length (10). The same study group in 2002, evaluated the 6-months retention of BWSTT in PD. Twenty-four patients with PD were randomized to receive BWSTT (20% of unweighting for 10 min + 10% of unweighting for 10 min + 0% of unweighting for an additional 10-min period) or CPT 3 times/week for 4 consecutive weeks. All patients were clinically evaluated at baseline and then monthly for 6 months. In this series, gait speed significantly improved in BWSTT respect to CPT only at month 1, while the improvement in the stride length was more marked in BWSTT group with respect to CPT and persisted until month 4 (11).

Toole et al. showed that 6-weeks of BWSTT increased gait speed and stride length, evaluated with clinical tests, and improved balance, measured with Computerized Dynamic Posturography. Of note, in this study no statistical difference in gait was observed when comparing patients treated with treadmill alone with patients treated with treadmill associated with weight-support (15).

In 2008, Fisher et al. speculated on the possible central mechanism responsible for clinical effects of BWSTT. Thirty subjects affected by PD were randomly assigned to three groups: high-intensity group (24 sessions of BWSTT), low-intensity group (24 sessions of CPT), zero-intensity group (8-weeks of education classes). Again, the high-intensity group improved the most at the end of treatment period, in particular in gait speed, step length, stride length, and double support. Of note, in this study a subgroup of patients was also tested with transcranial magnetic stimulation: in the BWSTT group Authors were able to record a lengthening of the cortical silent period, postulating that high-intensity training improved neuronal plasticity in PD, through BDNF and GABA modulation (25).

More recently, Rose et al. studied the efficacy of a high-intensity locomotor training in BWS condition achieved with a positive-pressure antigravity treadmill. When comparing training period (3 sessions/week for 8 weeks) with a control period (no intervention), they found a significant improvement in MDS-UPDRS (total and motor sub-scale) and walking distance (26). Indeed Ganesan et al. randomized 60 PD patients to 3 groups: (1) no specific exercise activity, (2) conventional gait training (30 min sessions, 4 times/week for 1 month), and (3) BWSTT (30 min sessions, 4 times/week for 1 month). At the end of a 4-weeks follow up, both intervention groups showed an improvement in the UPDRS score (total, motor, and sub-scores) and in gait parameters (walking distance, speed, and step length) when compared to non-exercising group; moreover, BWSTT appeared to be significantly superior respect to conventional gait training. At variance from the previous study, this latter one used an instrumental analysis of gait, which unfortunately was performed while the subjects were walking on the treadmill (instrumented 2-min walk test) and not during unassisted overground gait (23).

Lander and Moran studied the spatiotemporal gait effects of BWSTT with an instrumented 6-m device (GAITRite, CIR systems); they studied the effects of a single session of BWSTT in PD and healthy controls and showed an improved gait speed and cadence in both groups. Unfortunately, they did not investigate the effect of repetitive BWSTT sessions (24).

In the future, we hope that BWS might be improved and combined with novel technologies in order to develop new and individualized rehabilitative strategies. In this view, Park et al. combined BWS with a treadmill designed to adapt the walking speed according to the voluntary patient control via a feedback/feedforward control. Moreover, the environment around this BWSTT was enriched by a virtual reality system able to simulate real-life conditions. This approach proved safe and allowed the therapist to treat patients in more realistic overground gait conditions; indeed, the use of virtual obstacles, such as walls or narrow spaces, in the virtual reality setting allowed Authors to study freezing of gait in laboratory (27).

Another example is the combination of BWS with cues rehabilitation; Schlick et al. showed how BWSTT combined with visual cues was more effective in improving step length and gait symmetry when compared to an un-cued condition. It is also noteworthy that this multimodal approach was efficient and well-tolerated in advance PD patient with a V Hoehn and Yahr stage (28).

#### BWSTT Delivered With Robotic Devices

Ustinova et al. published the first positive case report on the short-term gait rehabilitation efficacy of BWSTT delivered to a PD patient with a robotic device (Lokomat-Hocoma Inc., Volketswil, Switzerland). The intervention consisted in a 2-weeks gait training, delivered 3 times per week, with each session lasting 90–120 min (29).

Lo et al. conducted a pilot study to assess the efficacy of BWSTT delivered with the Lokomat unit in reducing frequency of freezing of gait (FOG) in PD. Authors reported a 20% reduction in the average number of daily episodes of FOG and a 14% improvement in the FOG-questionnaire score (19).

In 2012, Picelli et al. enrolled 41 PD patients in the first randomized controlled study aimed to compare the efficacy of BWSTT delivered with a robot-assisted gait training (RAGT-gait Trainer GT1) to CPT (not focused on gait training) in improving gait in PD. They showed how RAGT was significantly superior respect to CPT in improving the 6-min walking test, the 10-meter walking test, stride length, single/double support ratio, Parkinson's Fatigue Scale and UPDRS score (20).

Carda et al. subsequently designed a randomized controlled study to assess superiority of robotic-gait training with BWS (Lokomat with 50% of unweighting for 15 min followed by 30% unweighting for an additional 15-min period) when compared to treadmill training without BWS. The Authors failed to record any significant differences between groups at the 6-min walking test, the 10-meter walking test and the Time up-and go test, although all the parameters significantly improved in both groups, with a positive effect persisting up to 6 months after rehabilitation (30).

In the paper published by Sale et al., the main aim was the comparison between a new end-effector robotic BWS device (G-EO system device) and treadmill training without weight-support. After 4-weeks of rehabilitation, the statistical analysis showed a significant improvement with the robotic intervention in gait speed, step length and stride length, but the between-group analysis was not statistically significant (21).

In 2013, Picelli et al. designed a comprehensive randomized controlled study aimed to compare robotic BWSTT (RAGT—gait Trainer GT1) with treadmill training without BWS (TT) and CPT. Sixty subjects with mild to moderate PD were enrolled and evaluated before treatment (T0), at the end of a 4-weeks rehabilitative programme (T1) and after 3 months (T2). This study failed to demonstrate the superiority of RAGT in improving gait speed when compared to TT; at variance, both RAGT and TT proved more effective than CPT as regards gait speed and walking capacity. It is worth noting that the improvement in gait speed was considered clinically significant (namely > 0.25 m/s at the 10-meter test) only after the RAGT approach (31).

Finally, Galli et al. compared the effects of BWS delivered with a robotic end effector (G-EO system) with TT not only on the spatio-temporal gait parameters, but also on the range of motion of the most important lower limb joints. The results showed that robotic rehabilitation produced an improvement in the kinematic gait profile at the proximal level (hip and pelvis) when compared to TT without BWS. These results are useful from a clinical point of view because they suggest that rehabilitation with BWS and robotic gait training could be recommended in specific sub-groups of PD patients (for example in those with a deficit of pelvis and hip mobility at baseline) (22).

## A Controlled Trial Comparing BWSTT With Overground Gait Training

### Materials and Methods

#### Subjects

Subjects were enrolled among consecutive PD patients hospitalized in the Neurorehabilitation Unit of the IRCCS Mondino Foundation of Pavia, Italy. Thirty-six patients affected by Idiopathic PD, according to the UK Brain Bank diagnostic criteria were included according to the following inclusion criteria:

- disease stage II–III Hoehn and Yahr (H&Y);
- stable dosage of dopaminomimetic drugs for 3 months before study enrollment.

Exclusion criteria were:

- moderate to severe cognitive impairment (MMSE ≤ 21);
- unpredictable motor fluctuations;
- moderate to severe orthopedic diseases or other pathological conditions (e.g., severe postural abnormalities) that might affect gait training.

During the observation period, no change was allowed to the dopaminomimetic drugs.

All participants gave their written informed consent for participation in the study, which was carried out according to the Declaration of Helsinki and was approved by the Ethics Committee. At the time of the approval of the protocol the local Ethics Committee was held at the IRCCS Mondino Foundation.

Demographic and clinical details of the subjects are shown in Table 1.

**Table 1:** Demographic and clinical characteristics of BWSTT and Control groups.

**Demographic and clinical characteristics**		**BWSTT group (*n* = 14)**	**Control group (*n* = 22)**	***p*-value**
Age (years)		71.9 ± 10.2	71.7 ± 7.5	0.958
Disease duration (years)		11.4 ± 11.4	10.18 ± 4.8	0.652
H&Y stage		2.3 ± 0.6	2.1 ± 0.4	0.479
UPDRS III score		33.4 ± 11.6	34.4 ± 12.0	0.810
FIM score		99.6 ± 12.3	99.6 ± 16.2	0.996
Sex	M	6 (42.9%)	12 (54.5%)	0.733
	F	8 (57.1%)	10 (45.5%)	
Disease onset	Right	2 (14.3%)	10 (45.5%)	0.107
	Left	6 (42.9%)	8 (36.4%)	
	Bilateral	6 (42.9%)	4 (18.2%)	
Patients with freezing (%)		4 (28.6%)	10 (45.5%)	0.485
Therapy	L	3 (21.4%)	4 (18.2%)	0.950
	L-DA	3 (21.4%)	5 (22.7%)	
	L-E	2 (14.3%)	2 (9.1%)	
	L-E-DA	6 (42.9%)	11 (50.0%)	

*L, levodopa; DA, dopaminoagonist; E, entacapone*.

### Procedures

**Figure 1:**
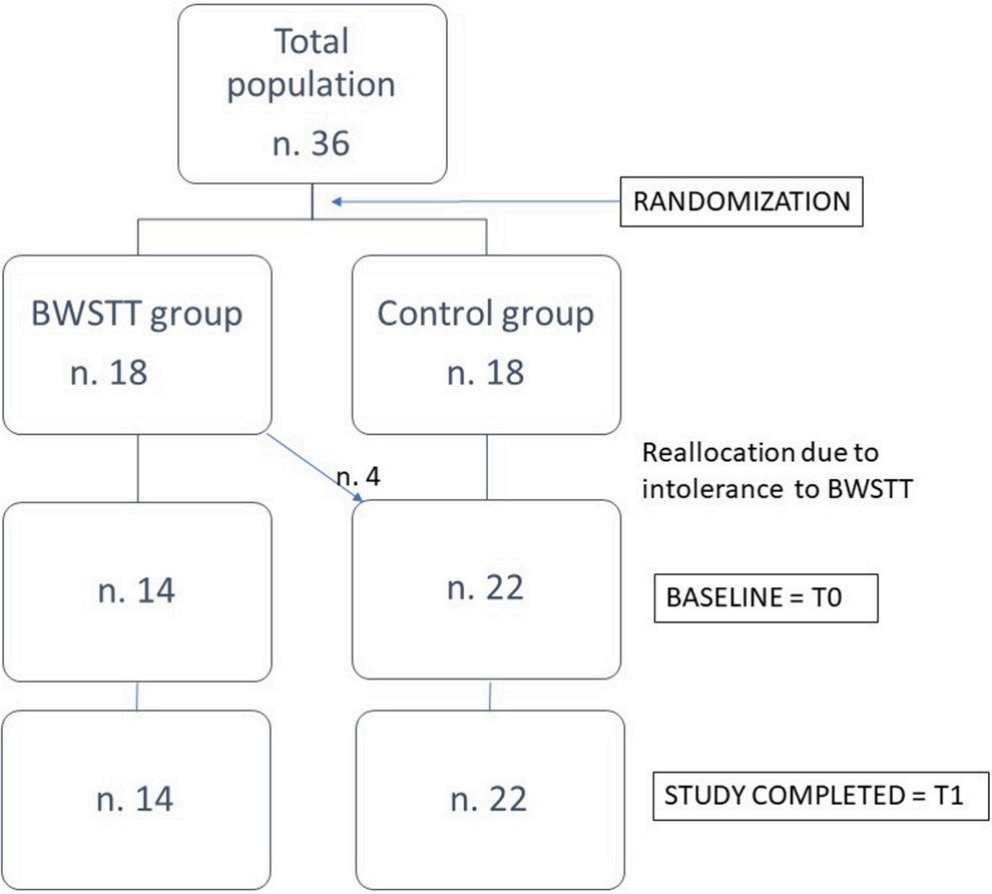
Study flow and patients' disposition.

Subjects were randomly assigned to two groups: 18 PD patients were assigned to the “BWSTT group” and 18 patients to the “Control group.” Before starting treatment, patients of BWSTT group performed a 20-min single session of BWSTT in order to test feasibility and tolerability. Four of them did not tolerate BWSTT: one patient reported an increase in his pre-existing hip pain, two patients with pre-existing spondyloarthrosis complained of low back pain, one patient reported that the procedure induced anxious symptoms. These 4 patients were re-allocated to the Control group, so that the final disposition of patients in the two groups was as follows: 14 patients (8 women and 6 men) in the BWSTT group and 22 patients (10 women and 12 men) in the Control group (Figure 1).

Patients in both groups underwent 5 daily rehabilitation sessions per week for 4 consecutive weeks. Both groups underwent daily 40-min sessions of traditional physical therapy (PT) followed by a 20-min session of overground gait training (Control group) or of gait training with BWSTT (BWSTT group).

#### Traditional Treatment

The traditional PT rehabilitation treatment included passive, active and active-assisted exercises, according to the methods commonly used (Kabat, Bobath) and previously published (32, 33).

Every 40-min treatment session consisted in isotonic and isometric exercises for the major muscles of the limbs and trunk including cardiovascular warm-up exercises (5 min), muscle stretching exercises (10 min), muscle stretching exercises for functional purposes (10 min), balance training exercises (10 min), relaxation exercises (5 min). This protocol was designed in accordance with PD rehabilitation guidelines and evidences in the literature (34).

#### Treatment With Body Weight Support Treadmill Training (BWSTT)

The sessions were conducted on a treadmill with partial weight unload. Specifically, the patient performed 10-min treadmill walk with a support corresponding to 20% of his/her own weight, followed by a 5-min rest and a second 10-min session on the treadmill with a support corresponding to 10% of his/her own weight. In the initial treadmill session, the starting speed of the treadmill was set to 0.5 km/h, subsequent increments of 0.5 km/h per minute were added to reach the maximum speed that was comfortably tolerated by the patient. This latter was used for the entire training period.

All patients were examined by a neurologist with expertise in movement disorders at the beginning of hospitalization (T0) and at the end of the neurorehabilitation period (+4 weeks, T1). The clinical assessment involved a complete neurological examination and administration of the following clinical scales, validated for the assessment of the disability:

- for the assessment of PD severity: the Unified Parkinson's Disease Rating Scale, part III (UPDRS-III) (35);
- for the assessment of functional independence: the Functional Independence Measure (FIM) (36).

### Computerized Gait Analysis

The instrumental assessment of gait was conducted at T0 and T1 by an experienced laboratory Technician using an Optokinetic Gait Analysis System associated to the software Myolab Clinic (ELITE, BTS Engineering, Milan), composed of six infrared cameras, with a sampling rate of 100 Hz. According to the Davis protocol, 21 spherical reflective markers (15 mm in diameter) were applied along the body. Synchronized data acquisition and data processing were performed by analyzer software (BTS, Milan, Italy). In order to perform kinematic analysis of gait, patients were instructed to walk at their preferred speed along a 10-meter walkway with the initial step on the side of disease onset. For each session, we acquired at least three performances and calculated the mean. In order to obtain the best individual performance, all recordings were conducted in the ON phase. The sessions were recorded at 5-min intervals to allow complete recovery from fatigue.

#### Data Analysis

After acquisition of the recordings, the video files were analyzed and the spatial-temporal variables were measured during all phases of gait cycle using the software Myolab Clinic (ELITE, BTS Engineering, Milan). We calculated the following variables:

- Speed (m/s);
- Cadence, expressed as the number of steps per minute (step/min);
- Stride duration (s);
- Stride length (m);
- Duration of stance expressed as a percentage of the duration of step (%);
- Duration of swing expressed as a percentage of the duration of step (%);
- Number of strides on a 10-meter distance.

### Statistical Analysis

The sample size and power of the study were calculated using the portal “Open Source Epidemiologic Statistics for Public Health” (www.openepi.com). We calculated the sample size with the following parameters: confidence interval (two sided) 95%; power 80%; difference between groups 20% (with a standard deviation between 20 and 25% for each group). The Statistical Package for the Social Sciences (SPSS) for Windows, version 21.0, was used for the calculation.

The distribution of each variable was evaluated using “skewness” and “kurtosis.” Moreover, the data were plotted using a “*Q*-*Q* plot” that confirmed normal distribution of all tested variables. For qualitative variables we used crosstabs analysis, performing statistical significance with Fisher exact test by case.

Quantitative variables are presented as mean values ± standard deviation. The main analysis regarding clinical scales and gait parameters was performed with the ANOVA (analysis of variance) for repeated measures with two factors: (1) Time (two levels: T0 vs. T1); (2) Group (two levels: BWSTT vs. Control).

We also performed a sub-analysis to asses intra-group variations between T0 and T1 using *t*-test for paired samples; delta percentage variations between groups (BWS vs. controls) were tested using a *t*-test for independent samples. The level of significance was set for convention at a *p* < 0.05, always corrected if necessary.

## Results

The demographic and clinical characteristics of the two groups are shown in Table 1. No statistically significant differences were found between groups at baseline. Clinical features and gait parameters of each subject at T0 and T1 are presented in Tables 2–3 for Control and BWSTT groups, respectively.

**Table 2:** Clinical features and gait parameters (T0 and T1) for each patient in Control group.

**ID**	**Age**	**Disease duration (years)**	**Freezing**	**Disease onset**	**Therapy**	**H&Y**	**UPDRS**		**FIM**		**Speed (m/s)**		**Cadence (step/min)**		**Stride duration (ms)**		**Stride length (m)**		**Stance (%)**		**Swing (%)**		**N**^****°****^ **strides (10 m)**	
							**T0**	**T1**	**T0**	**T1**	**T0**	**T1**	**T0**	**T1**	**T0**	**T1**	**T0**	**T1**	**T0**	**T1**	**T0**	**T1**	**T0**	**T1**
1	70–75	11	Y	Le	L-E-DA	2	28	28	110	110	0.87	1.32	69.6	105.5	1623.2	1137.0	0.70	0.70	67.7	62.0	32.3	38.0	5.0	5.0
2	70–75	7	Y	Le	L-DA	2	40	29	89	95	0.90	0.79	77.2	80.5	1587.2	1389.2	0.59	0.68	66.6	66.4	33.4	33.6	4.0	4.0
3	55–60	6	N	R	L-E-DA	2	39	27	90	104	0.96	1.22	81.3	78.0	1644.2	1538.0	0.66	0.77	67.4	68.3	32.6	31.7	4.0	4.0
4	70–75	6	Y	Le	L	2	44	41	89	94	0.82	0.60	91.5	77.4	1311.0	1551.0	0.50	0.44	76.5	68.8	23.5	31.2	7.0	8.0
5	55–60	5	N	R	L-E	2	11	5	108	126	1.00	1.00	88.6	97.2	1354.0	1377.0	0.62	0.70	65.5	63.0	34.6	37.0	4.0	4.0
6	65–70	14	Y	R	L-E-DA	2	50	43	92	107	0.43	0.49	87.0	83.6	1500.0	1435.7	0.30	0.33	72.6	71.8	27.4	28.1	11.5	10.7
7	70–75	21	Y	Le	L-DA	2	57	44	44	55	0.28	0.50	65.3	74.6	1838.7	1608.3	0.24	0.38	71.1	69.0	28.9	31.0	14.5	9.0
8	60–65	20	N	Le	L-E-DA	2	38	29	105	111	0.77	0.96	79.8	86.7	1561.9	1384.6	0.56	0.62	58.4	47.9	41.5	53.5	6.0	5.5
9	65–70	14	Y	R	L-E-DA	3	50	43	92	107	0.43	0.50	110.8	110.2	1500.0	1435.7	0.30	0.33	62.5	61.9	37.5	38.1	12.0	10.5
10	80–85	7	N	Le	L	2	33	24	96	114	0.58	0.48	70.8	75.3	1699.0	1594.5	0.46	0.35	62.7	67.8	37.3	32.2	8.0	10.0
11	75–80	6	N	R	L-DA	2	57	44	44	55	0.35	0.53	75.1	79.6	1599.0	1508.0	0.26	0.37	61.0	63.7	39.0	36.3	13.5	9.5
12	70–75	10	N	Le	L-DA	2	28	23	105	119	1.16	1.25	115.8	134.7	1036.8	891.3	0.56	0.52	64.6	63.2	35.4	36.8	6.0	6.5
13	70–75	6	N	B	L-E-DA	2	38	29	105	111	0.75	1.07	75.9	94.0	1598.5	1314.0	0.56	0.64	60.4	48.3	39.6	51.8	6.5	5.5
14	75–80	8	Y	B	L-E-DA	3	36	32	109	117	0.82	0.82	81.9	88.3	1467.5	1359.3	0.56	0.52	68.2	69.8	31.8	30.3	6.0	6.5
15	80–85	7	N	R	L	2	29	20	112	121	0.88	1.17	80.8	102.0	1300.5	1178.8	0.61	0.64	65.0	64.8	35.0	35.2	6.0	5.5
16	70–75	10	Y	B	L-E-DA	2	15	10	121	129	0.83	0.95	98.5	94.6	1219.0	1269.5	0.47	0.56	67.5	63.5	32.5	36.5	7.5	6.0
17	75–80	9	N	B	L-E-DA	3	28	22	128	128	0.33	0.31	84.2	86.2	1468.7	1392.3	0.23	0.20	69.6	62.7	30.4	37.3	15.3	19.0
18	75–80	13	Y	R	L-E	2	40	21	100	123	0.88	0.82	85.9	78.8	1401.5	1523.0	0.43	0.58	55.3	58.5	44.7	41.5	6.0	6.0
19	70–75	7	N	R	L-E-DA	2	22	18	92	109	0.77	1.15	92.2	95.7	1304.8	1255.3	0.44	0.67	57.1	64.2	42.9	35.8	7.5	5.0
20	55–60	20	N	Le	L-E-DA	2	33	30	96	105	0.87	0.88	85.0	97.8	1226.3	1240.5	0.48	0.51	61.6	27.3	38.4	72.7	7.5	7.0
21	80–85	8	Y	R	L	2	15	10	105	113	0.50	0.50	99.6	70.9	1109.5	1695.5	0.28	0.39	65.4	68.2	34.6	31.8	12.5	9.0
22	80–85	9	N	R	L-DA	3	34	26	109	116	0.66	0.71	83.1	75.8	1452.5	1485.5	0.48	0.52	63.5	61.8	36.5	38.2	7.5	6.8

*Le, left; R, right; B, bilateral, L, levodopa; DA, dopaminoagonist; E, entacapone*.

**Table 3:** Clinical features and gait parameters (T0 and T1) for each patient in BWSTT group.

**ID**	**Age**	**Disease duration (years)**	**Freezing**	**Disease onset**	**Therapy**	**H&Y**	**UPDRS**		**FIM**		**Speed (m/s)**		**Cadence (step/min)**		**Stride duration (ms)**		**Stride length (m)**		**Stance (%)**		**Swing (%)**		**N**^****°****^ **strides (10 m)**	
							**T0**	**T1**	**T0**	**T1**	**T0**	**T1**	**T0**	**T1**	**T0**	**T1**	**T0**	**T1**	**T0**	**T1**	**T0**	**T1**	**T0**	**T1**
23	75–80	12	N	Le	L-E-DA	3	23	14	104	106	0.92	1.00	88.1	96.2	1466.0	1348.8	0.51	0.58	60.5	60.6	39.5	39.4	6.0	6.0
24	65–70	2	N	Le	L-DA	3	46	32	125	111	0.76	0.97	78.5	96.9	1528.5	1238.5	0.54	0.56	60.2	58.7	39.8	41.3	6.5	6.0
25	40–45	9	Y	Le	L-E	2	21	13	109	121	0.75	0.86	71.8	82.1	1675.7	1461.5	0.48	0.58	54.4	54.0	45.6	46.0	6.0	6.0
26	80–85	7	N	R	L-DA	2	28	21	100	111	0.80	0.85	77.7	94.6	1548.8	1275.0	0.53	0.50	70.7	67.2	29.3	32.8	6.0	7.0
27	65–70	23	Y	R	L-E-DA	2	35	22	100	111	0.56	0.73	77.9	77.7	1543.5	1549.8	0.40	0.52	59.3	60.4	40.7	39.6	9.0	6.5
28	70–75	38	N	B	L-E-DA	2	52	28	98	103	1.06	0.90	105.4	107.3	1144.0	1124.3	0.48	0.47	59.7	61.4	40.3	38.6	6.0	7.5
29	70–75	10	N	B	L-E-DA	2	41	31	103	119	0.36	0.52	57.3	77.1	2104.3	1569.0	0.35	0.38	70.1	59.7	29.9	40.3	10.0	9.0
30	75–80	2	N	B	L	2	18	17	104	108	0.57	0.48	81.7	71.9	1470.3	1672.3	0.39	0.48	69.2	72.3	30.8	27.7	9.0	9.5
31	60–65	14	Y	B	L-E-DA	2	33	30	91	105	0.90	1.18	106.9	113.6	1122.5	1056.8	0.47	0.58	64.6	59.0	35.4	41.0	7.5	6.0
32	75–80	1	N	B	L	2	24	21	85	94	0.63	0.67	82.6	88.4	1462.3	1359.3	0.43	0.43	69.6	67.4	30.3	32.6	8.0	8.0
33	75–80	4	N	Le	L	2	36	28	100	102	0.53	0.54	71.9	73.3	1668.5	1638.5	0.41	0.41	53.9	64.4	46.1	35.6	8.5	8.5
34	70–75	1	N	Le	L-E-DA	3	21	9	102	113	0.55	1.03	86.3	98.4	1129.8	1221.0	0.29	0.58	60.3	59.7	39.7	40.3	12.0	6.0
35	85–95	7	N	Le	L-DA	2	37	18	103	114	0.69	0.63	87.8	86.0	1368.5	1397.0	0.44	0.41	64.6	65.9	35.4	34.1	8.0	8.5
36	70–80	30	Y	B	L-E	3	53	43	70	80	0.53	0.55	117.3	113.1	1023.7	1063.3	0.25	0.52	66.8	68.2	33.2	31.8	14.0	13.0

*Le, left; R, right; B, bilateral; L, levodopa; DA, dopaminoagonist; E, entacapone*.

### Clinical Scales

We found a significant effect of factor “Time” for both UPDRS-III score [*F* = 102.857; *df*_(1, 34)_; *p* = 0.001] and FIM score [*F* = 63.222; *df*_(1, 34)_; *p* = 0.001], without any significant interaction for factors “Group” and “Time^*^Group” (Table 4).

**Table 4:** Effect of BWSTT (BWSTT group) and traditional rehabilitation (Control group) on clinical scales and kinematic variables of gait.

**Variables**	**BWSTT group (*****n*** **= 14)**		**Control group (*****n*** **= 22)**		**ANOVA for repeated measures**		
	**T0**	**T1**	**T0**	**T1**	**Time**	**Group**	Time^*^Group
UPDRS	33.4 ± 11.5	23.3 ± 9.1	34.4 ± 12.0	26.8 ± 10.6	*F* = 102.857	*F* = 0.467	*F* = 2.029
					*df* (1, 34)	*df* (1, 34)	*df* (1, 34)
					***p*** **= 0.001**	*p* = 0.499	*p* = 0.163
FIM	99.5 ± 12.3	107.0 ± 10.4	99.6 ± 16.2	110.1 ± 15.5	*F* = 63.222	*F* = 0.019	*F* = 1.720
					*df* (1, 34)	*df* (1, 34)	*df* (1, 34)
					***p*** **= 0.001**	*p* = 0.892	*p* = 0.198
Speed (m/s)	0.68 ± 0.1	0.78 ± 0.2	0.72 ± 0.2	0.82 ± 0.3	*F* = 11,306	*F* = 0.224	*F* = 0.003
					*df* (1, 34)	*df* (1, 34)	*df* (1, 34)
					***p*** **= 0.002**	*p* = 0.639	*p* = 0.956
Cadence (step/min)	85.9 ± 16.4	90.7 ± 14.1	86.0 ± 16.4	89.0 ± 17.0	*F* = 6.233	*F* = 0.026	*F* = 0.272
					*df* (1, 34)	*df* (1, 34)	*df* (1, 34)
					***p*** **= 0.018**	*p* = 0.872	*p* = 0.606
Stride duration (ms)	1447.2 ± 283.3	1353.3 ± 210.4	1438.0 ± 198, 3	1392.0 ± 251.0	*F* = 43,741	*F* = 0.064	*F* = 0.268
					*df* (1, 34)	*df* (1, 34)	*df* (1, 34)
					***p*** **= 0.036**	*p* = 0.802	*p* = 0.608
Stride length (m)	0.41 ± 0.1	0.49 ± 0.1	0.46 ± 0.1	0.51 ± 0.1	*F* = 17.700	*F* = 0.544	*F* = 0.617
					*df* (1, 34)	*df* (1, 34)	*df* (1, 34)
					***p*** **= 0.001**	*p* = 0.466	*p* = 0.438
Stance (%)	63.0 ± 5.6	62.6 ± 5.0	64.4 ± 5.7	61.2 ± 11.1	*F* = 1.879	*F* = 0.66	*F* = 1.191
					*df* (1, 34)	*df* (1, 34)	*df* (1, 34)
					*p* = 0.179	*p* = 0.799	*p* = 0.283
Swing (%)	36.9 ± 5.6	37.3 ± 5.0	35.5 ± 5.7	38.7 ± 11.2	*F* = 1.926	*F* = 0.058	*F* = 1.231
					*df* (1, 34)	*df* (1, 34)	*df* (1, 34)
					*p* = 0.174	*p* = 0.811	*p* = 0.275
N° strides (10 m)	8.2 ± 2.2	7.6 ± 1.8	8.1 ± 3.0	7.6 ± 3.5	*F* = 3.364	*F* = 0.078	*F* = 0.001
					*df* (1, 34)	*df* (1, 34)	*df* (1, 34)
					*p* = 0.065	*p* = 0.781	*p* = 0.970

*ANOVA for repeated measures with two factors: (1) Time (T0 vs. T1) and (2) Group (BWSTT vs. Control). Bold text highlights significant results*.

In the intra-group sub-analysis, the UPDRS-III score significantly decreased at T1 in both BWSTT group (T0 = 33.4 ± 11.5, T1 = 23.3 ± 9.1; T0 vs. T1 *p* = 0.01) and Control group (T0 = 34.4 ± 12.0, T1 = 26.8 ± 10.6; T0 vs. T1 *p* = 0.01). In line with this result, also FIM significantly improved at T1 in both groups of patients (BWSTT group: T0 = 99.5 ± 12.3, T1 = 107.0 ± 10.4; *p* = 0.01; Control group: T0 = 99.6 ± 16.2, T1 = 110.1 ± 15.5; *p* = 0.01).

### Gait Parameters

The main analysis showed a significant effect of factor “Time” for speed [*F* = 11,306; *df*_(1, 34)_; *p* = 0.002], cadence [*F* = 6.233; *df*_(1, 34)_; *p* = 0.018), stride duration [*F* = 43, 741; *df*_(1, 34)_; *p* = 0.036] and stride length [*F* = 17.700; *df*_(1, 34)_; *p* = 0.001]. Factor “Group” and the interaction “Time^*^Group” were not significant for all gait parameters (Table 4).

The intragroup sub-analysis showed a significant increase in speed (from 0.6 ± 0.1 to 0.7 ± 0.2 m/s; T0 vs. T1 *p* = 0.01), cadence (from 85.9 ± 16.4 to 90.7 ± 14.1; T0 vs. T1 *p* = 0.01), length of stride (from 0.41 ± 0.1 m to 0.59 ± 0.1 m; T0 vs. T1 *p* = 0.04) and with a reduction in the duration of stride (from 1447.2 ± 283.3 to 1353.3 ± 210.4 ms; T0 vs. T1 *p* = 0.01) and in the number of strides (from 8.2 ± 2.2 to 7.6 ± 1.8 for 10-meters; T0 vs. T1 *p* = 0.02) (Table 5) at T1 in the BWSTT group.

**Table 5:** *Post-hoc* analysis of intragroup changes in gait parameters for BWSTT and Control groups from baseline (T0) to the end of the rehabilitative period (T1).

**Variables**	**BWSTT group (*****n*** **= 14)**			**Control group (*****n*** **= 22)**		
	**T0**	**T1**	***p*-value T1 vs. T0**	**T0**	**T1**	***p*-value T1 vs. T0**
Speed (m/s)	0.68 ± 0.1	0.78 ± 0.2	**0.001**	0.72 ± 0.2	0.82 ± 0.3	**0.001**
Cadence (step/min)	85.9 ± 16.4	90.7 ± 14.1	**0.001**	86.0 ± 16.4	89.0 ± 17.0	0.151
Stride duration (ms)	1447.2 ± 283.3	1353.3 ± 210.4	**0.001**	1438.0 ± 198.3	1392.0 ± 251.0	0.143
Stride length (m)	0.41 ± 0.1	0.59 ± 0.1	**0.046**	0.46 ± 0.1	0.51 ± 0.1	0.130
Stance (%)	63.0 ± 5.6	62.6 ± 5.0	0.577	64.4 ± 5.7	61.2 ± 11.1	**0.022**
Swing (%)	36.9 ± 5.6	37.3 ± 5.0	0.575	35.5 ± 5.7	38.7 ± 11.2	**0.022**
N° strides (10 m)	8.2 ± 2.2	7.6 ± 1.8	**0.022**	8.1 ± 3.0	7.6 ± 3.5	0.111

*Bold text highlights significant results*.

In the Control group, at T1 we observed a significant increase in speed (from 0.7 ± 0.2 to 0.8 ± 0.3 m/s; T0 vs. T1 *p* = 0.01) and swing duration (from 35.5 ± 5.7 to 38.7 ± 11.2%; T0 vs. T1 *p* = 0.02), with a consequent reduction of stance duration (from 64.4 ± 5.7 to 61.2 ± 11.1%; T0 vs. T1 *p* = 0.02) (Table 5).

**Figure 2:**
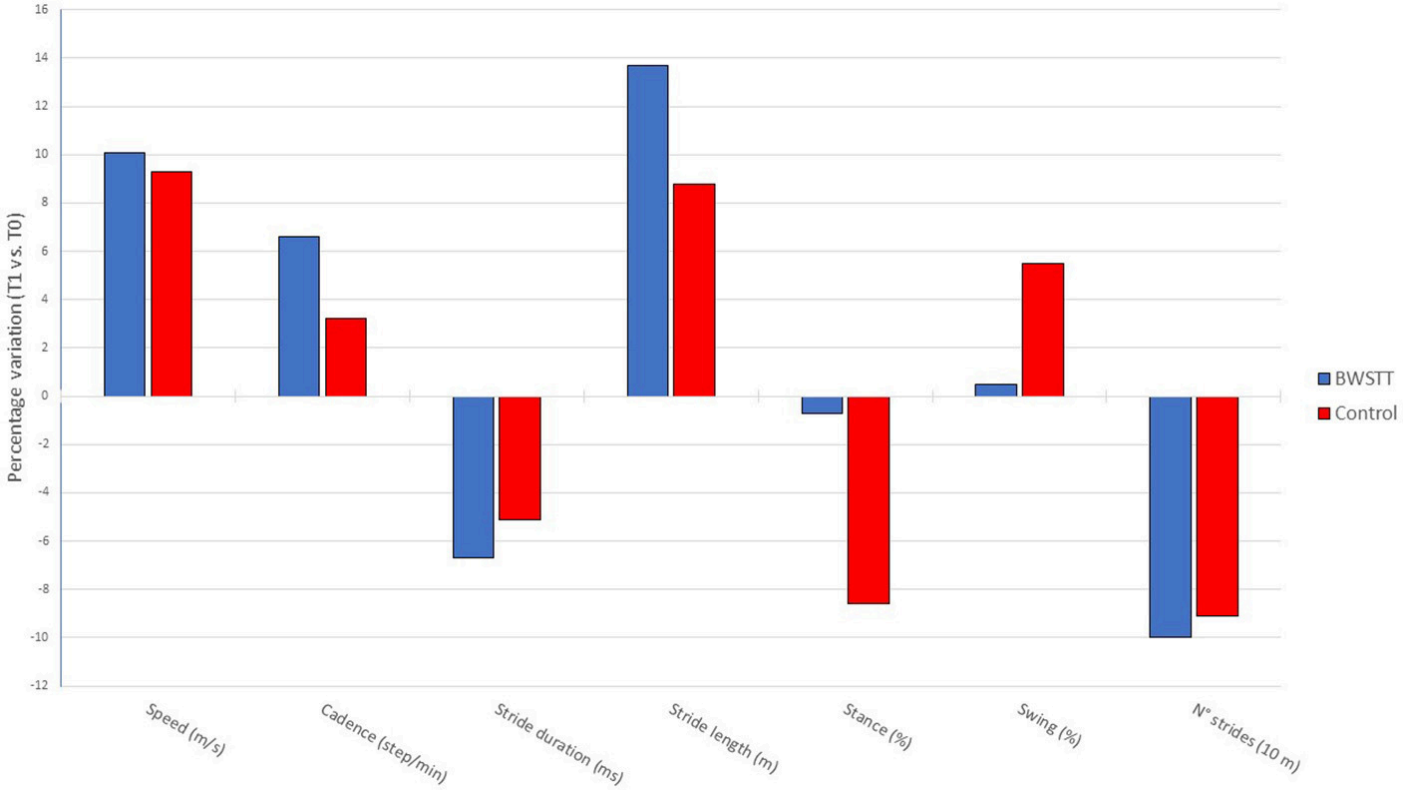
Comparison of delta % changes in gait parameters from baseline (T0) to the end of the rehabilitative period (T1) in BWSTT and Control groups.

When comparing the percent change of the gait parameters induced by the two rehabilitation interventions, no statistically significant differences were found in any gait parameters between Control group and BWSTT group (Figure 2).

## Discussion

Gait disorders represent common and disabling symptoms in PD. In recent years, multidisciplinary rehabilitation treatment has acquired a key role in improving the motor function (34). Effectiveness of BWSTT in improving gait, balance and motor function evaluated with both clinical scales and instrumental Kinematic Computerized Gait Analysis has been demonstrated in neurological diseases as stroke (12) and spinal cord injuries (13). Previous findings suggested that Treadmill Training (TT) could improve gait parameters in PD patients (16), but data available from the literature on the specific effect of BWSTT in the rehabilitation of gait of PD subjects do not allow conclusive inference. In the literature we can find some reviews on the topic, but the strength of their conclusions is partially limited by methodological bias. In particular, these reviews poorly differentiate between treadmill training used alone, treadmill training plus BWS, or BWS delivered with robotic devices (16, 37), which actually represent quite different modalities of rehabilitation from a methodological point of view.

To the best of our knowledge this is the first controlled study that evaluates the effect of a 4-weeks rehabilitative program with a non-robotic BWSTT using instrumentally-recorded gait parameters in PD patients during overground, spontaneous gait. BWSTT was compared with standard rehabilitation and it induced an improvement in the score of the clinical scales, namely UPDRS and FIM, thus confirming the clinical efficacy reported in previous studies (10, 11, 16, 18). The main analysis of our study showed a significant improvement in multiple parameters of gait at the end of the 4-weeks rehabilitative program in both study groups. In this view, BWSTT did not prove to be more efficient than conventional overground gait training. At the intragroup sub-analysis, however, BWSTT showed a specific profile of improvement upon the kinematic gait parameters recorded with a 6-camera optoelectronic system for CGA. Indeed, at the end of the 4-weeks rehabilitation period only the BWSTT group experienced a significant increase in the stride length, stride duration and cadence, together with a reduction in the number of strides and in the duration of stride. The group receiving standard rehabilitation protocol showed solely a percent reduction in the stance phase of the step with a percent increase in the swing phase.

Different mechanisms may be hypothesized to explain the clinical efficacy of BWSTT on gait and balance. In stroke patients, treadmill training reduces cardiovascular demands and energy expenditure (12, 14). Aerobic training is however not the main driver of the effect, since treadmill training alone resulted in a worse outcome than BWSTT in stroke (12, 14). In the case of PD, a possible role may be played by the impact on BWSTT on baroreflex sensitivity (38), defined as a measure of sensitivity of the cardiac limb of the baroreflex and derived from the change in inter-beat interval for unit change in systolic pressure (39, 40). Indeed, low blood pressure variation and a decrease in baroreflex sensitivity significantly contribute to orthostatic hypotension in PD (41). Ganesan et al. demonstrated that 4-weeks of BWSTT significantly improve baroreflex sensitivity in patients with PD and prevent orthostatic blood pressure fall (38). No conclusive data about gait are available in literature on the comparison between BWSTT and TT in PD. Both treatments seem to improve gait, either when used alone or as an add-on to standard rehabilitation treatment (16). Some Authors have hypothesized a neuromechanical effect of BWSTT, involving central pattern generators (CPGs). CPGs are load and sensory dependent and BWSTT may alter the supraspinal and spinal influences on CPG neuromotor activity (42, 43). Evidence suggests that an abnormal proprioception and dysfunctional sensorimotor integration is frequent in PD. In PD patients, external stimuli are able to modulate the motor pattern. Acoustic, visual and somatosensory cues help the patients to start and maintain a rhythmic motor task (44–46). Each type of cue activates a different pattern of supraspinal motor control. In particular, sensory cues enable the voluntary dorsolateral pre-motor control system, bypassing the supplementary motor area's deficit that alters automatic movement (45). In this frame, it is intuitive that treadmill training, with and without BWS, does represent a symmetrical and repetitive sensory stimulus, capable of increasing the rhythm of motion, and increasing the discharge activity of additional locomotor areas (45). However, in BWSTT, the mechanism could be more complex, leading to an implementation of the activity also on the latter circuit. Studies with near infra-red spectroscopy (NIRS) showed, in fact, that treatment with BWSTT induced an increase in activation of the primary and supplementary motor areas in PD patients (47). Other possible explanations of the effectiveness of BWSTT on gait rehabilitation include specific motor learning of the task, improvement in postural reflexes (48), neural plasticity induced by physical activity and dependent activity (neurogenesis, synaptogenesis and molecular adaptation) (49, 50) and the normalization of corticomotor excitability/cortical reorganization, especially in the supplementary motor area in subjects with PD (25). Another possible explanation for the beneficial changes observed following BWSTT is the improvement of balance as suggested by previous reports (18). Unfortunately, our study was not designed to investigate balance.

While the two gait training approaches under investigation showed a different profile of improvement as regards the various gait parameters, the statistical analysis failed to detect significant changes when evaluating the impact of BWSTT and classic overground rehabilitation (in term of percent changes) upon the different gait parameters. As a consequence, it is fair to state that, in terms of efficacy, both treatments may be proposed for gait rehabilitation in PD patients. What can drive the selection of BWSTT over overground gait training is the safety of the procedure. Indeed, the BWS harness practically prevents falls in patients during the rehabilitative session. This allows physiotherapists to focus more properly on the different segments involved in walking, instead of having to worry about steading the subject. This means that BWSTT may find its ideal indication in the more severely affected PD patients. This impression obviously needs scientific confirmation in specific trials. In this frame, it is important to note that our findings suggest that BWSTT is safe, but may not be well-tolerated by patients with chronic pain and anxious symptoms. Therefore, it is advisable to conduct an initial BWSTT test session in order to assess tolerability before starting treatment.

## Limitations

This study was conducted on a small number of patients who may not be representative of the full extension of the functional disability that is associated to PD. Furthermore, due to the above described tolerance issues, the 2 arms were not perfectly balanced in terms of numerosity. However, though these considerations may limit the transferability of the present data to the general PD population we feel that it is important to report our experience in full because of its useful implication in the real life setting.

In this study we did not evaluate the effect of BWSTT on balance, which may represent one of the components that affect the positive results recorded on the gait performance in our patients. An additional arm, evaluating the effect of treadmill alone, would have increased the possibility to dissect out the role of different components. Further studies are needed to evaluate the effect of BWSTT on orthostatic hypotension and balance.

## Conclusions

In conclusion, our results show that BWSTT and standard traditional rehabilitation treatment are both effective in improving clinical motor functions and kinematic gait parameters in PD. BWSTT showed some interesting peculiarities in its profile of gait improvement. This observation, together with the well-known specific technical features of the procedure suggests that BWSTT treatment may find a preferential indication for gait training in PD patients with moderate or severe postural instability, balance disorders, orthostatic hypotension.

## Author Contributions

EB designed the protocol and drafted the manuscript. RD designed the protocol, performed the statistical analysis, and revised the manuscript. MA, SC, and CP enrolled patients. CD and MF acquired data for the paper and organized the database. GS and CT provided substantial contributions to the conception of the protocol and critically revised the manuscript.

### Conflict of Interest Statement

The authors declare that the research was conducted in the absence of any commercial or financial relationships that could be construed as a potential conflict of interest.
